# Don’t stand so close to me: psychopathy and the regulation of interpersonal distance

**DOI:** 10.3389/fnhum.2013.00907

**Published:** 2014-01-10

**Authors:** Joana B. Vieira, Abigail A. Marsh

**Affiliations:** ^1^Department of Psychology, Georgetown UniversityWashington DC, United States; ^2^Faculty of Medicine, University of PortoPorto, Portugal; ^3^Laboratory of Neuropsychophysiology, Faculty of Psychology and Educational Sciences, University of PortoPorto, Portugal

**Keywords:** psychopathy, coldheartedness, interpersonal distance, approach/avoidance, amygdala

## Abstract

Psychopathy is characterized by callous and unemotional personality traits, such as reduced empathy and remorse, and a tendency toward deviant interpersonal behaviors. It has been suggested that subtle behavioral cues in individuals with high levels of psychopathic traits may betray their personality during interpersonal interactions, but little research has addressed what these clues might be. In this study, we investigated whether psychopathic traits predict interpersonal distance preferences, which have been previously linked to amygdala functioning. 46 healthy participants performed a behavioral task in which the distance they preferred to maintain between themselves and an experimenter was measured across a series of trials. Psychopathic traits, including Coldheartedness, Fearless Dominance, and Self-centered Impulsivity were assessed using the Psychopathic Personality Inventory-Revised (Lilienfeld and Widows, 2005). Results demonstrated that Coldheartedness predicted preferred interpersonal distance, with more coldhearted participants preferring shorter distances. These findings suggest that interpersonal distance preferences may signal psychopathic traits, particularly callousness, supporting accounts of amygdala dysfunction in psychopathy.

## INTRODUCTION

Psychopathy is a personality variable characterized by callous and unemotional personality traits, such as lack of empathy and guilt, and antisocial behavioral tendencies, such as impulsiveness and aggression (Frick and White, 2008; Feilhauer and Cima, 2013). Given highly psychopathic individuals’ penchant for deviant interpersonal behaviors, a troublesome feature of psychopathy is that individuals’ outward appearance rarely betrays their affective and interpersonal deficits (Cleckley, 1988). Identifying observable behavioral cues that signal high levels of psychopathy would be highly desirable. That such cues exist is suggested by research showing observers can reliably detect psychopathic features from small samples, or “thin slices,” of behavior (Fowler et al., 2009), although little evidence yet exists concerning what these cues might be.

In this study, we examined whether psychopathy predicts preferred interpersonal distance. The regulation of interpersonal distance appears to be supported by the amygdala. It was recently shown that a patient with selective bilateral amygdala lesions (SM) reported an abnormal lack of discomfort when standing very close to an experimenter and preferred interpersonal distances that were significantly shorter than the average preferred distance of controls (Kennedy et al., 2009). In the same study, fMRI results revealed that amygdala activity in healthy individuals was modulated by interpersonal distance, with activation increasing when subjects knew an experimenter was standing close to the scanner. This is consistent with research in animals showing that the amygdala is involved in regulating approach and avoidance behaviors, such that monkeys with selective amygdala lesions show reduced avoidance of novel or naturally threatening objects (Machado et al., 2009), other monkeys in dyadic interactions (Emery et al., 2001), and human strangers (Mason et al., 2006).

These findings are in agreement with the possibility of aberrant interpersonal distance regulation in psychopathy, as robust evidence links psychopathy, particularly its affective component, to amygdala dysfunction. Both functional and structural amygdala abnormalities have been reported in high psychopathy scorers (Birbaumer et al., 2005; e.g., Kiehl et al., 2001; Gordon et al., 2004; Yang et al., 2010; Ermer et al., 2012; Marsh and Cardinale, 2014). Moreover, striking similarities between the behavior of psychopathic individuals and amygdala lesion patients have been observed. Both populations show facial, vocal, and postural fear recognition impairments (Adolphs et al., 1994; Scott et al., 1997; Blair et al., 2002; Munoz, 2009), reduced subjective experience of fear (Feinstein et al., 2011; Marsh et al., 2011), reduced startle modulation (Angrilli et al., 1996; Syngelaki et al., 2013), reduced anticipatory skin conductance response (Patrick et al., 1994; Bechara et al., 1995), and deficient aversive conditioning (LaBar et al., 1995; Rothemund et al., 2012).

No previous studies have investigated whether psychopathic traits affect the regulation of interpersonal distance during social interactions. Research on motoric approach/avoidance to social stimuli using a computer joystick task showed that high psychopathy scorers display less avoidance of social threats (angry faces; Von Borries et al., 2012), but it is unknown whether this would extend to actual interpersonal distance regulation during social interactions. To investigate this question, we used a paradigm based on that developed by Kennedy et al. (2009) to assess interpersonal distance preferences in a community sample varying in psychopathy. In line with evidence showing amygdala dysfunction in psychopathy, we hypothesized that psychopathic traits would predict a preference for shorter interpersonal distances, paralleling what was observed with SM (Kennedy et al., 2009). Moreover, we were interested in investigating which psychopathic traits were most closely associated with interpersonal distance preferences. Recent reports linked reduced amygdala responsiveness specifically to the callous and unemotional components of psychopathy (White et al., 2012; Viding et al., 2013; Sebastian et al., 2014). Using the Psychopathic Personality Inventory-Revised (PPI-R; Lilienfeld and Widows, 2005), it has been demonstrated that Coldheartedness, which is associated with callousness, reduced empathy, and guiltlessness (Gaughan et al., 2009; Seibert et al., 2011), is associated with reduced amygdala activity to experimentally manipulated fearful faces (Han et al., 2011). In line with this evidence, we predicted that PPI-R Coldheartedness scores would most accurately predict preferred interpersonal distance.

## MATERIAL AND METHODS

### PARTICIPANTS

Forty-six participants (17 male; *M* age = 20.47, *SD* = 2.2, range 18–25) were recruited from the Georgetown University community and compensated for their participation. All participants reported not having any prior psychiatric or neurologic diagnoses, history of brain injuries or substance abuse, and not taking any psychotropic medication at the time of the study. The study was approved by the Institutional Review Board at Georgetown University, and all participants provided informed written consent in accordance with the Declaration of Helsinki.

### PSYCHOPATHY MEASURES

Psychopathy was assessed using the PPI-R (Lilienfeld and Widows, 2005), a self-report instrument designed to measure psychopathic traits in a dimensional manner. This is consistent with the idea that psychopathy is a set of traits continuously distributed in the general population rather than a clinical taxon (Krueger et al., 2005; Skeem et al., 2011), and, like other personality disorders, it can be more reliably assessed using dimensional models of personality (Miller et al., 2001; Marcus et al., 2004). Although it was developed to assess psychopathic traits in community samples, the PPI-R and its predecessor correlate with psychopathy measures predominantly used in institutionalized samples, such as the PCL-R (Poythress et al., 2010). The PPI-R contains 154 items organized in eight subscales: social influence, fearlessness, stress immunity, Machiavellian egocentricity, rebellious non-conformity, blame externalization, carefree non-planfulness, and coldheartedness. These subscales, with the exception of coldheartedness, load into two higher-order factors, PPI-I or fearless dominance, and PPI-II or Self-centered impulsivity. Fearless dominance scores index interpersonal dominance and low anxiety (e.g., “When I’m in a frightening situation, I can “turn off” my fear almost at will”), and Self-centered impulsivity scores are related to disinhibition and impulsive behavior (e.g., “I like to act first and think later”; Lilienfeld and Widows, 2005; Gaughan et al., 2009). Coldheartedness seems to be largely independent of both these factors, and is therefore regarded simultaneously as a subscale and a higher-order dimension (Skeem et al., 2011). Coldheartedness scores index callousness and lack of sympathy for others (e.g., “When someone is hurt by something I say or do, that’s their problem”; Lilienfeld and Widows, 2005; Gaughan et al., 2009).

### INTERPERSONAL DISTANCE TASK

Following Kennedy et al. (2009), interpersonal distance preferences were measured across 32 trials for each participant, divided in two blocks. In the experimenter-walking block, participants began by standing with their toes on a mark on the floor. The experimenter stood four meters away and then began walking toward the participant at a natural gait (approximately 1 m/s). Participants were instructed to tell the experimenter to stop at their preferred distance (i.e., the distance at which they felt “the most comfortable”). This was then fine-tuned by allowing the participant to ask the experimenter to move slightly forward or back after she had stopped. Distance between the chins of the experimenter and participant was then measured using a digital laser tape measure (Bosch, model DLR130). In the participant-walking block, participants walked toward the experimenter and stopped upon reaching their preferred distance, also followed by adjustments after stopping if they desired. The order of the blocks was counterbalanced across participants. Within each block, trials varied in terms of *eye contact* (with or without) and *approach/withdrawal* (in approach trials the participant or experimenter started apart and walked forward, in avoidance trials they started close, with their toes approximately 2–3 cm apart, and walked backward). This resulted in eight different trial types, each repeated four times, with the order of trials being randomized within each block.

All participants were tested in the same room by two experimenters, one who gave task instructions and made distance measurements, and another who walked or stood. The experimenter who walked/stood maintained a neutral facial expression throughout the task and refrained from showing any signs of discomfort upon approaching or being approached by the participant.

## RESULTS

**FIGURE 1:**
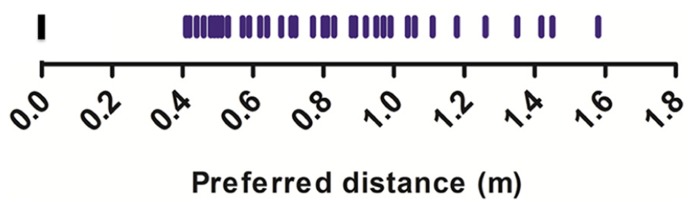
**Average preferred distances (m) from the experimenter**.

We calculated the average preferred distance per participant, across and for each trial type. The distribution of values obtained across trials ranged from 0.41 to 1.58 m (*M* = 0.80, *SD* = 0.30; **Figure 1**), a distribution very similar to that reported by Kennedy et al. (2009) using the same task (*t*(32) = 0.45, *p* = 0.65).

Exploration of PPI-R total and factor scores revealed only minor deviations from normality, namely in the symmetry of the distributions (**Table 1**). Skewness values were < 1.0 for all variables and thus not indicative of significant deviations from normality (Field, 2005; Blanca et al., 2013). In order to be conservative, however, we log-transformed PPI-R total and factor scores and ran the analyses using both raw and log-transformed variables. These transformations did not affect the significance of any effects, so we present results obtained using non-transformed values in order to facilitate interpretation of findings.

**Table 1:** Sample characterization in terms of psychopathy scores (PPI-R total and factor scores).

	*Cronbach’s α*	*M (SD*)	Min–Max
PPI-Total	0.95	303.79 (44.69)	228–426
Coldheartedness	0.83	29.78 (6.94)	17–52
Fearless dominance	0.94	128.41 (24.98)	63–171
Self-centered impulsivity	0.92	145.40 (24.10)	105–223

We first examined the associations between overall preferred distance and PPI-R total and factor scores. Results revealed that overall preferred distance was only significantly associated only with Coldheartedness scores, with higher scorers preferring shorter distances (**Table 2**).

**Table 2:** Correlation coefficients indexing the associations between overall preferred distance (m) and PPI-R total and factor scores.

	PPI-R total	C	FD	SCI
Overall preferred distance	-0.11	-0.29^*^	-0.07	-0.05

* *p* < 0.05. C = Coldheartedness; *FD* = Fearless Dominance; SCI = Self-centered Impulsivity.

**FIGURE 2:**
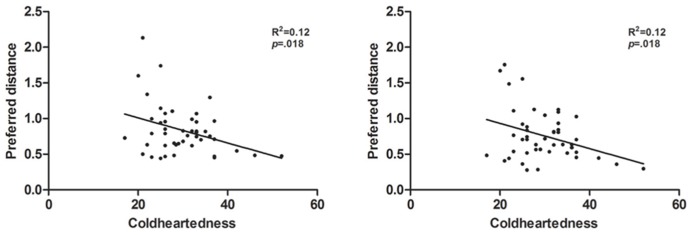
**Scatter plots depicting the association between Coldheartedness scores and preferred distance in approach trials with eye contact, in the experimenter (left) and participant-walking (right) blocks**.

Follow-up correlations with preferred distance in each trial type showed that the strongest associations between Coldheartedness and distance were obtained in approach trials with eye contact, irrespective of person walking (experimenter-walking: *r* = -0.35, *p* = 0.018; participant-walking: *r* = -0.35, *p* = 0.018; **Table 3**; **Figure 2**), although the difference between the magnitude of the correlation coefficients across trial types was not significant.

**Table 3:** Correlation coefficients indexing the associations between preferred distance (m) in each trial type and PPI Coldheartedness scores.

	Experimenter walking				Participant walking			
	Approach eye contact	Approach no eye contact	Withdrawal eye contact	Withdrawal no eye contact	Approach eye contact	Approach no eye contact	Withdrawal eye contact	Withdrawal no eye contact
C	-0.35^*^	-0.28	-0.27	-0.21	-0.35^*^	-0.28	-0.22	-0.07
FD	-0.04	-0.06	-0.1	-0.01	-0.15	-0.00	-0.20	-0.10
SCI	-0.05	-0.03	-0.05	-0.02	-0.09	-0.06	-0.05	-0.01

* *p* < 0.05. C = Coldheartedness; FD = Fearless Dominance; SCI = Self-centered Impulsivity.

To confirm the link between preferred distance and Coldhearted ness, we computed the average preferred distance in approach trials with eye contact by collapsing experimenter-walking and participant-walking trials, and performed linear regression analysis with the three PPI-R factors as predictors, and co-varying out age, sex, and the match between experimenter and participant sex. Results revealed that Coldheartedness was the only significant predictor of preferred distance, with higher scores associated with preference for shorter distances (**Table 4**). Consistent effects were obtained using the overall preferred distance as dependent variable (**Table 4**).

**Table 4:** Regression analysis results.

Preferred distance in approach trials with eye contact	B	Wald Chi-Square (1 df)	*p*
Coldheartedness	**–0.021**	**6.53**	**0.01**
Self-Centered Impulsivity	0.001	0.33	0.95
Fearless Dominance	0.000	0.00	0.57
**Overall preferred distance**			
Coldheartedness	**–0.016**	**4.68**	**0.03**
Self-Centered Impulsivity	0.001	0.34	0.56
Fearless Dominance	0.000	0.01	0.93

*Significant results highlighted in bold.*

To rule out possible effects of cultural variability within our sample, we repeated the correlation analyses after excluding subjects who were not born in the United States (*n* = 10). We also replicated the regression analyses with the total sample, adding nationality as a predictor of no interest. All observed associations between Coldheartedness and preferred distance remained significant.

**FIGURE 3:**
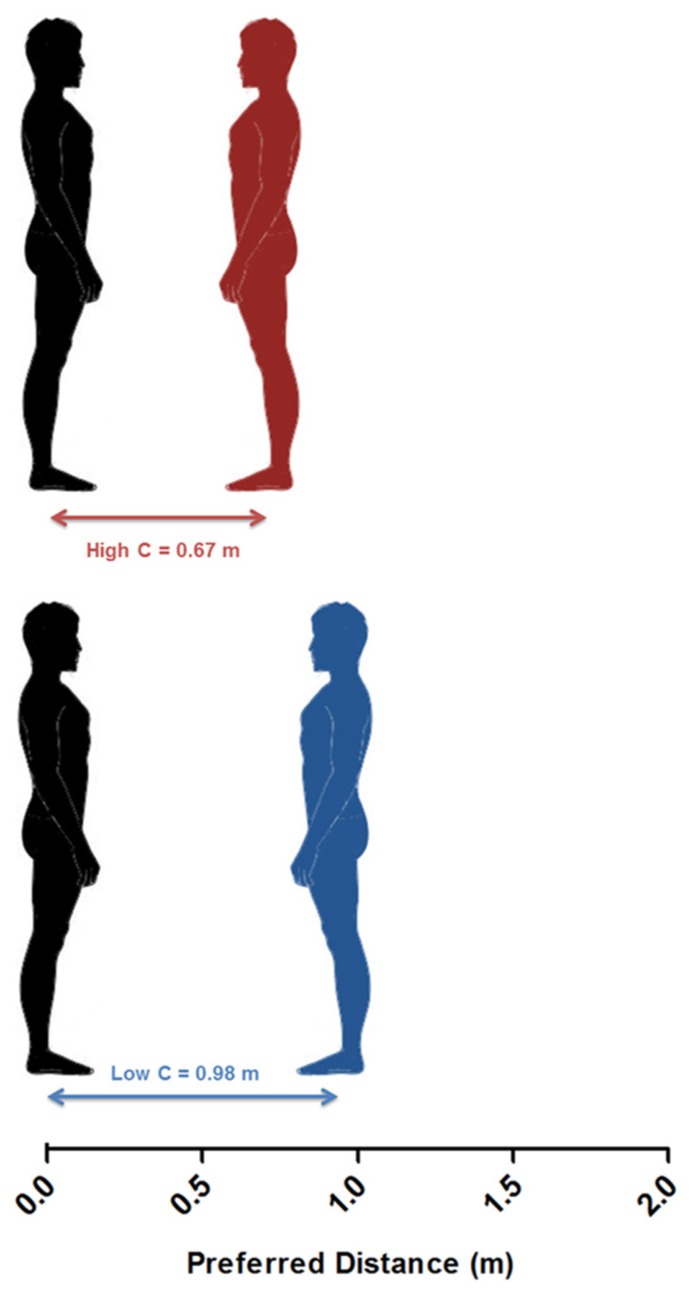
**Schematic representation (drawn to scale) of the average preferred distance of the highest (red) and lowest (blue) quartiles of Coldheartedness scorers**.

Finally, we split the sample into quartiles according to Coldheartedness scores to compare the average preferred distance in approach – eye contact trials between highest and lowest scorers. *T*-test results (*t*(24) = 2.04, *p* = 0.053; *d* = -0.78) showed that the highest quartile of Coldheartedness scorers (*M* = 0.67 m, *SD* = 0.21) preferred shorter interpersonal distances than the lowest quartile (*M* = 0.98 m, *SD* = 0.52; **Figure 3**), with the middle quartiles showing intermediate values (*M* = 0.71 m, *SD* = 0.23; *M* = 0.80 m, *SD* = 0.14).

## DISCUSSION

This study investigated whether psychopathic traits influence the distance individuals prefer to maintain between themselves and others in social interactions. Consistent with our hypothesis, results showed that PPI-R coldheartedness scores, which index interpersonal callousness (Gaughan et al., 2009), significantly predicted preferred distance, with more callous participants showing a preference for shorter distances. These patterns persisted even after potentially confounding variables, such as cultural background and sex, were accounted for in the analysis.

In his seminal work, Cleckley (1988) described psychopathy as a profound affective deficit that results in impaired patterns of interpersonal functioning. Nonetheless, he believed that individuals with psychopathic traits display a “mask of sanity” that gives them an appearance of normality, charm, and good intelligence. Fowler et al. (2009) argued that despite this “mask,” psychopaths’ lack of insight into their own deficits leads them to give away clues about their personality during interpersonal interactions. These clues, mainly non-verbal, may be picked up even from thin slices of behavior, and used by lay observers to make relatively reliable and accurate assessments of psychopathy, although the question of how these assessments are achieved remained unanswered by their study. Our results suggest that one clue that predicts the presence of heightened psychopathic traits, particularly callousness, is preferred interpersonal distance.

By demonstrating an association between coldheartedness and interpersonal distance regulation, a mechanism previously shown to be under the control of the amygdala (Kennedy et al., 2009), our results support the association between callous personality traits and amygdala dysfunction. This is in line with previous research linking atypical amygdala function to these traits more than to other features of psychopathy (Patrick et al., 1993; Marsh et al., 2008; Jones et al., 2009; White et al., 2012). Moreover, it is consistent with recent studies that specifically linked the PPI-R coldheartedness subscale to abnormal amygdala activity in response to social stimuli (Han et al., 2011).Finally, these results are in line with prior reports that psychopathic traits are associated with reduced avoidance of social stimuli (Von Borries et al., 2012). It should be noted, however, that contrary to Von Borries et al. (2012) our effects were specific for callous traits and not overall psychopathy. Moreover, we did not assess approach/avoidance behavior to threatening social stimuli. Despite these methodological differences, the fact that our results and those reported by Von Borries et al. (2012) were in the same direction further supports the association between psychopathic traits, especially callousness, and an atypical pattern of social approach/avoidance, which may extend to both threatening and non-threatening interactions. Our confidence in the present findings also relies on our sample size, which was larger than that used in the studies conducted by Kennedy et al. (2009), Han et al. (2011), and Von Borries et al. (2012), and yielded results that were in accordance with those studies.

One of the questions arising from our findings is why highly callous individuals would prefer to stand closer to other people, and whether this preference is related to other interpersonal behavioral patterns associated with psychopathy. Our trial-specific findings may help to address this question. Results showed that coldheartedness best predicted preferred distance when subjects were approached by or approached another person. These experimental conditions are the ones that best approximate a real-life aggressive encounter, suggesting that perhaps the regulation of interpersonal distance in highly callous individuals may relate to their demonstrated propensity for aggression in general and, particularly, for instrumental aggression (e.g., Viding et al., 2009; Walsh et al., 2009; Thornton et al., 2012). Although the link between personal space (i.e., the area maintained around oneself in social interactions; Sommer, 1959), and aggression has been investigated previously (e.g., Curran et al., 1978; Walkley and Gilmour, 1984), the personality traits mediating such association have never been systematically explored. It seems reasonable to assume that, for two people facing one another, shorter interpersonal distances would facilitate aggression by putting individuals within arm’s reach. Interestingly, the average frontal arm reach – around 90 cm for males and 82 cm for females (Parker et al., 1996) – is shorter than the preferred distance of low coldheartedness scorers, but longer than the preferred distance of high coldheartedness scorers in our study, suggesting that more callous participants tended to prefer distances that put the experimenter within their reach. In light of these data, we speculate that interpersonal distance preferences of highly callous individuals may mediate the relationship between callous traits and aggression, by producing behaviors that facilitate aggressive behavior. Although plausible and consistent with demonstrations that in high psychopathy scorers the decreased avoidance of threatening social stimuli is correlated with levels of instrumental aggression (Von Borries et al., 2012), this interpretation requires further testing. Furthermore, it would be important to confirm whether high and low coldheartedness scorers are equally able to detect if they are or not within the reach of another person to validate this interpretation.

An alternate explanation of our findings relates to the practical realization that those who maintain shorter interpersonal distances put themselves within others’ reach and, therefore, become more vulnerable to aggression. In fact, classical accounts of personal space assigned it a protective function against potentially unpleasant or threatening situations (Dosey and Meisels, 1969). In addition to instrumental aggression, Von Borries et al. (2012) found an association between decreased social avoidance and inability to experience personal distress in psychopathic individuals. This interpretation is consistent with previous reports of reduced fearfulness in high psychopathy scorers (e.g., Marsh et al., 2011), which has been linked to atypical amygdala function in this population. In sum, it is possible that preference for shorter interpersonal distances in highly callous individuals reflects deficient social avoidance mechanisms, which could result from deficits in fear experience as a consequence of amygdala dysfunction. However, our results are not entirely congruent with this interpretation, given that we did not find any significant effects of the PPI fearless dominance component (which among other traits indexes fearlessness) on preferred interpersonal distance.

In sum, this study demonstrated that the callousness component of psychopathy is associated with a preference for maintaining shorter distances in social interactions, a pattern that is likely to be linked to abnormal amygdala functioning. By identifying an observable behavior that potentially signals high callous traits, this study adds a novel finding to the literature concerning interpersonal behavior in psychopathy. In future research, the inclusion of additional measurements in the same paradigm, such as assessing the participant’s walking pace and the number of adjustments necessary to choose the preferred distance, as well as explicitly manipulating the threat level and familiarity of the interactions, would enable a more precise characterization of the interpersonal behavioral styles associated with psychopathic traits. Furthermore, given prior research demonstrating a relationship between psychopathic traits and difficulties in recognizing emotional expressions of distress (e.g., Blair et al., 2001), it would be relevant to investigate whether interpersonal distance preferences in highly callous individuals are associated with difficulties in identifying signs of discomfort in others, or with the disregard of those signs. Even though it is not likely that our findings were driven by these potential deficits (as we tried to minimize displays of discomfort by the experimenter during the task), the possible relation between emotional recognition deficits and interpersonal distance preferences in individuals varying in psychopathic traits should be investigated in future studies. Finally, further research is needed to address the putative associations between interpersonal distance preferences, fear, and aggression as a function of psychopathic personality traits, and to directly investigate its neural basis.

## Conflict of Interest Statement

The authors declare that the research was conducted in the absence of any commercial or financial relationships that could be construed as a potential conflict of interest.
